# Serum 25-Hydroxyvitamin D Concentrations at Birth in Children Screened for HLA-DQB1 Conferred Risk for Type 1 Diabetes

**DOI:** 10.1210/jc.2018-02094

**Published:** 2019-01-16

**Authors:** Marjaana Mäkinen, Eliisa Löyttyniemi, Maarit Koskinen, Mari Vähä-Mäkilä, Heli Siljander, Mirja Nurmio, Juha Mykkänen, Suvi M Virtanen, Olli Simell, Heikki Hyöty, Jorma Ilonen, Mikael Knip, Riitta Veijola, Jorma Toppari

**Affiliations:** 1 MediCity, University of Turku, Turku, Finland; 2 Department of Pediatrics, University of Turku and Turku University Hospital, Turku, Finland; 3 Department of Biostatistics, University of Turku, Turku, Finland; 4 Children’s Hospital, University of Helsinki and Helsinki University Hospital, Helsinki, Finland; 5 Institute of Biomedicine, Research Centre for Integrative Physiology and Pharmacology, University of Turku, Turku, Finland; 6 Research Centre of Applied and Preventive Cardiovascular Medicine, University of Turku, Turku, Finland; 7 Public Health Promotion Unit, Department of Public Health Solutions, National Institute for Health and Welfare, Helsinki, Finland; 8 Faculty of Social Sciences/Health Sciences, University of Tampere, Tampere, Finland; 9 Tampere Center for Child Health Research, Tampere University and University Hospital and Science Center, Tampere University Hospital, Tampere, Finland; 10 Department of Virology, Faculty of Medicine and Life Sciences, University of Tampere, Tampere, Finland; 11 11Fimlab Laboratories, Pirkanmaa Hospital District, Tampere, Finland; 12 Immunogenetics Laboratory, Institute of Biomedicine, University of Turku, Turku, and Clinical Microbiology, Turku University Hospital, Turku, Finland; 13 Research Programs Unit, Diabetes and Obesity, University of Helsinki, Helsinki, Finland; 14 Folkhälsan Research Center, Helsinki, Finland; 15 Department of Pediatrics, PEDEGO Research Unit, Medical Research Center Oulu, University of Oulu, Oulu, Finland; 16 Department of Children and Adolescents, Oulu University Hospital, Oulu, Finland

## Abstract

**Context:**

Vitamin D has several effects on the immune system that might be of relevance for the pathogenesis of type 1 diabetes (T1D).

**Objective:**

To evaluate whether umbilical cord serum concentrations of 25-hydroxy-vitamin D (25[OH]D) differ in children developing either islet autoimmunity (IA) or overt T1D during childhood and adolescence.

**Design:**

Umbilical cord serum samples from 764 children born from 1994 to 2004 with HLA-DQB1 conferred risk for T1D participating in the Type 1 Diabetes Prediction and Prevention Study were analyzed for 25(OH)D using an enzyme immunoassay.

**Setting:**

DIPP clinics in Turku, Oulu, and Tampere University Hospitals, Finland.

**Participants:**

Two hundred fifty children who developed T1D diabetes at a median age of 6.7 years (interquartile range [IQR] 4.0 to 10.1 years) and 132 additional case children who developed IA, *i.e.,* positivity for multiple islet autoantibodies. Cases were matched for date of birth, gender, and area of birth with 382 control children who remained autoantibody negative. The median duration of follow up was 9.8 years (IQR 5.7 to 13.1 years).

**Main Outcome Measure:**

The median 25(OH)D concentrations.

**Results:**

The median 25(OH)D concentration in cord serum was low [31.1 nmol/L (IQR 24.0 to 41.8); 88% <50 nmol/L], but not statistically different between children who developed T1D or IA and their control groups (*P* = 0.70). The levels were associated mainly with geographical location, year and month of birth, age of the mother, and maternal intake of vitamin D during pregnancy.

**Conclusions:**

The 25(OH)D concentrations at birth are not associated with the development of T1D during childhood.

## Teaser

Umbilical cord serum samples were measured for 25[OH]D in 382 case children developing either islet autoimmunity or T1D, and in 382 matched controls. The levels were low and not associated with T1D.

Vitamin D is one of the essential players in metabolic and physiological processes in the human body. It has multiple effects on the immune system and its role in the pathogenesis of immune-mediated diseases has long been suspected. We noticed an increase in 25-hydroxyvitamin D (25[OH]D) concentrations in Finnish children after year 2003, when vitamin D fortification of milk started in Finland, which preceded the plateauing of the rapid increase in type 1 diabetes (T1D) incidence that had continued for more than 50 years (1). However, we found no difference in median 25(OH)D concentration between children who developed T1D and healthy matched control children when the children were observed from the age of three months until the diagnosis of T1D (2).

T1D is an immune-mediated disease, which can manifest at any age (3). Diagnosis in early childhood, before the age of four years, is quite common especially in Finland, where the incidence is highest in the world (4) and some children develop T1D even before the age of one year (5). The factors initiating or triggering the immune-mediated process leading to the disease must be operative before that, perhaps already during fetal life. Some changes can be seen early on and we have detected differences in the lipidomic profile in cord serum samples in children who later progressed rapidly to T1D, but not in those developing islet autoimmunity (IA) without progressing to overt disease (6).

Here we assessed the possible association of fetal vitamin D levels and the development of IA and progression to clinical T1D by measuring umbilical cord serum 25(OH)D concentration in the Type 1 Diabetes Prediction and Prevention Study (DIPP) birth cohort study. Furthermore, we analyzed maternal intake of vitamin D during pregnancy in a subcohort.

## Materials and Methods

The study population comprised children (born between 1994 and 2004), who participated in DIPP in Finland. The DIPP project is an ongoing population-based prospective birth cohort study aimed at exploring means to predict and prevent progression to clinical type 1 diabetes (T1D) (7). Briefly, newborn infants with HLA-DQB1–conferred susceptibility to T1D are recruited from the University Hospitals in Turku (located at 60 °N), Oulu (located at 65 °N), and Tampere (located at 61 °N), Finland. The children attend the study centers for follow-up visits at 3- to 12-month intervals and their serum samples are analyzed for T1D-related autoantibodies. Islet cell antibodies (ICA) were used as the primary screening tool for *β*-cell autoimmunity. If a child seroconverted to positivity for ICA, all the preceding and subsequent samples of this child were analyzed for insulin autoantibodies (IAA), antibodies to the 65 kDa isoform of glutamic acid decarboxylase (GADA), and to the tyrosine phosphatase-related islet antigen 2 (IA-2A). Data from the autoantibody-negative children participating in the follow-up were collected until they were 15 years of age or until the end point of this study (July 2016). Data from the autoantibody-positive children were collected until the follow-up for this study ended or until they were diagnosed with T1D according to the World Health Organization criteria, whichever came first.

We analyzed the concentration of 25-hydroxyvitamin D (25[OH]D) in umbilical cord serum samples from 764 DIPP study participants with a nested case control design. There were 133 case control pairs in the Turku cohort, 137 in the Oulu cohort, and 112 in the Tampere cohort. Case children comprised two groups; the majority of the cases (250 children) were diagnosed with T1D by the end of July 2016 (T1D+) and the minority of cases (132 children) showed IA testing positive for multiple (≥2) autoantibodies but not progressing to overt T1D by the end of the study period (IA+). The cases were included based on the availability of samples and one control subject was selected for each case child. All control subjects (T1D− and IA−) remained autoantibody-negative and nondiabetic throughout the follow-up, and they were pairwise matched for age (birth within 30 days), gender, and study center.

Mixed arterial/venous umbilical cord blood was collected in the delivery room and these samples were used for both serum extraction and genetic screening. Serum samples were stored at −70°C. All children were initially screened for HLA-DQB1 alleles (8). An extended six-scale HLA-DR/DQ genotype-based T1D risk classification (9) was available in 625 of the 764 children. The three groups with decreased or neutral risk were combined into a nonincreased risk group, as the number of children in these groups was small. One child (IA+) carried a genotype conferring strongly decreased risk, 6 children (2 IA− and 4 T1D−) had genotypes conferring slightly decreased risk, and 28 children (7 IA+, 10 IA−, 2 T1D+, and 9 T1D−) carried genotypes conferring neutral risk, so that the nonincreased risk group included 35 children, whereas there were 135 children (30 IA+, 28 IA−, 33 T1D+, and 44 T1D−) with slightly increased risk, 279 children (71 IA+, 29 IA−, 125 T1D+, and 54 T1D-) with moderately increased risk, and 176 children (23 IA+, 16 IA−, 89 T1D+, and 48 T1D−) with genotypes conferring highly increased risk for T1D.

We were able to obtain maternal dietary data collected by a validated food frequency questionnaire after the birth of the child. The mothers were asked for food consumption during one month preceding the pregnancy leave, *i.e.,* the eighth month of pregnancy. Total energy intake and vitamin D intake from food, supplements, and in total were used in the current study, as described by Marjamäki *et al.* (10), from the mothers of 363 children in this study (63 IA+, 60 IA−, 121 T1D+, and 119 T1D−); 168 in the Oulu and 195 in the Tampere cohorts.

A commercial immunoassay kit (Immunodiagnostic Systems Ltd, Boldon, UK) was used for the 25(OH)D analyses, as previously described (1). The intra-assay coefficient of variation was 6.5% and the sensitivity was 5 nmol/L. The performance target set by the Vitamin D External Quality Assessment Scheme Advisory Panel for 25(OH)D assays was met (11).

Statistical analyses were performed with JMP Pro 12.0.1 and SAS for Windows version 9.4 (SAS Institute, Cary, NC) using multiway analysis of variance and Fisher exact test for categorical variables. Season of birth and study center were used as adjusting factors in all analysis. The value of *P* < 0.05 (two-tailed) was taken to indicate statistical significance. The year was divided into seasons as in our previous studies (1, 2), *i.e.,* winter (January to March), spring (April to June), summer (July to September), and fall (October to December). The 25(OH)D concentrations, energy intake and vitamin D intake from food, supplements and in total, were log-transformed responses in analyses requiring normal distribution. As zero values cannot be log transformed, 0.1 µg was added to vitamin D intake values from supplements to enable the log transformation and analysis. Possible confounding factors were controlled for by adding background variables to the statistical models as described in the Results section.

The current study was conducted according to the guidelines of the Declaration of Helsinki and was approved by the Joint Commission on Ethics of Turku University and Turku University Central Hospital. Written informed consent was obtained from all guardians of the subjects.

## Results

**Figure 1.**
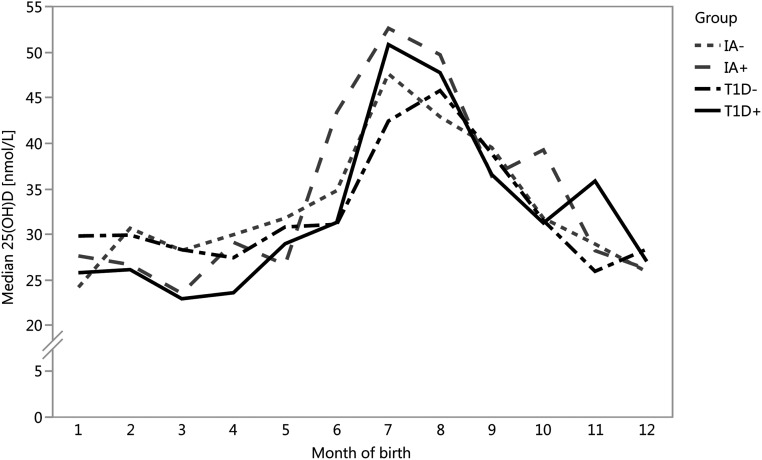
Monthly distribution of median 25(OH)D concentrations in cord serum samples were similar in the four groups. There were no statistically significant differences between T1D+ (solid black line) and T1D− (dashed black line) children (*P* = 0.39) or between IA+ (dashed gray line) and IA− (short-dashed gray line) children (*P* = 0.63). Serum 25(OH)D concentrations were statistically significantly affected by month of birth (*P* = 0.002).

Altogether, the study cohort included 382 case (250 T1D+, 132 IA+) and 382 control (250 T1D−, 132 IA−) children, who were matched for age, gender, and study center, as described. Serum 25(OH)D concentrations were statistically significantly affected by month of birth (Fig. 1) (*P* = 0.002) and study center (*P* = 0.03), so that the children of the Oulu center (the most northern of the of the centers located at 65°N) had substantially lower levels than the children in Turku (60°N) and Tampere (61°N) centers, but not by gender(*P* = 0.64). As dates of birth of cases and their controls were matched within one month, the age difference between them was small, the median being 6.5 days (IQR 2 to 13 days) for T1D+ and T1D−, and 6 days (IQR 3 to 15) for IA+ and IA− participants. The children were born rather evenly around the year. There were 210 children born in the winter (62 T1D+, 63 T1D−, 44 IA+, and 41 IA−); 182 children born in the spring (64 T1D+, 61 T1D-, 28 IA+, and 29 IA−); 187 born in the summer (59 T1D+, 62 T1D−, 33 IA+, and 33 IA−); and 185 born in the fall (65 T1D+, 64 T1D−, 27 IA+, and 29 IA−).

The basic characteristics were similar between the study groups T1D+, T1D−, IA+, and IA− (Table 1). As anticipated (12), the T1D+ children seroconverted to autoantibody positivity at an earlier age than IA+ children, and the follow-up time was shorter in the T1D+ group because of the design of the study.

**Table 1.** General Characteristics of the Study Population

	T1D+ N = 250	T1D− N = 250	IA+ N = 132	IA− N = 132	*P*
25(OH)D, nmol/L	30.7 (22.9– 43.3)	31.1 (24.5–39.8)	30.7 (23.3–41.8)	31.2 (25.2–40.5)	0.70
Weight, kg	3.59 (3.26–3.90)	3.65 (3.30–3.97)	3.64 (3.26–3.97)	3.63 (3.33–3.90)	0.76
Height, cm	50 (49–52)	51 (49–52)	51 (49–52)	50 (49–52)	0.58
Seroconversion age, y	2.0 (1.1–4.0)	NA	4.0 (1.8–6.2)	NA	**<0.001**
Age at T1D diagnosis, y	6.7 (4.0–10.1)	NA	NA	NA	
Gestational age, wk	39.9 (38.7–40.7)	40.1 (39.0–40.9)	39.9 (38.7–40.7)	40 (38.9–41)	0.32
Age of mother, y	30.1 (26.9–33.9)	29.2 (26.4–32.7)	29.8 (26.0–34.8)	29.8 (26.8–33.7)	0.45
Time of follow-up, y	6.2 (3.4–9.3)	10.5 (6.9–14.3)	12.6 (9.1–14.8)	12.0 (8.4–14.7)	**<0.001**

Values are median (IQR). The value of *P* < 0.05 (two-tailed) was taken to indicate statistical significance and is marked in bold.

Most importantly, there were no statistically significant differences in the median 25(OH)D concentrations in cord serum between the study groups (*P* = 0.70). The 25(OH)D concentrations of 675 cord serum samples were below 50 nmol/L and thus 88% of the study children had suboptimal (13) vitamin D levels. When months were combined to seasons of birth, its significance on 25(OH)D concentrations increased (from *P* = 0.002 to *P* < 0.0001), with a peak concentration during the summer months (Fig. 1).

The association between 25(OH)D concentrations and group differences (T1D+, T1D−, IA+, IA−) was studied with multiway ANOVA. Also, these analyses were adjusted by season and study center. All interactions between the independent explanatory variables were evaluated and if they were nonsignificant they were removed from the final model. In addition, the effect of year of birth, HLA-DQB1 group, HLA-DR/DQ group, number of antibodies, ponderal index, and nutritional factors with 25(OH)D concentration were evaluated in separate models with the analysis method explained above. Differences between the groups were nonsignificant in all the models reported below.

The median age at the diagnosis of T1D was 6.7 years (IQR 4.0 to 10.9 years). The median age at seroconversion to autoantibody positivity in the T1D+ group was 2.0 years (IQR 1.1 to 4.0) (n = 235, information was not available for 15 subjects) and 4.0 years (IQR 1.8 to 6.2) in the IA+ group (n = 132). The 25(OH)D concentrations were not associated with the age at T1D diagnosis (*P* = 0.53) nor with the age of seroconversion to autoantibody positivity (*P* = 0.72).

During the follow-up there were 146 T1D+ and 31 IA+ children who tested positive for all four autoantibodies, 64 T1D+ and 45 IA+ children positive for three autoantibodies, 22 T1D+ and 56 tested maximally positive for two autoantibodies, and additional 6 T1D+ children tested positive for the maximum of one autoantibody in their sample series, and 12 T1D+ children had unknown autoantibody status (*i.e.,* incomplete set of samples available for antibody analysis) before the diagnosis of T1D. There were no statistically significant differences in 25(OH)D concentrations between these autoantibody groups (*P* = 0.38). The situation was similar when only biochemical autoantibodies were considered as were the numbers of children involved; there were 146 T1D+ and 31 IA+ children with three biochemical autoantibodies, 65 T1D+ and 46 IA+ with two, 24 T1D+ and 55 IA+ children with one, and three T1D+ children with only ICA, *i.e.,* zero biochemical autoantibodies, all having similar 25(OH)D concentrations (*P* = 0.32).

There were 11 IA+ and 86 T1D+ children with IAA as the first appearing persistent biochemical autoantibody. No differences were found in 25(OH)D concentrations (*P* = 0.61) between the groups of cases and their matched 97 controls in this subcohort. Similar results were obtained in the 70 IA+ and 39 T1D+ children in whom GADA was the first appearing persistent autoantibody; no differences were seen between the cases and their 109 controls (*P* = 0.51).

HLA-DQB1 group (moderate risk, high risk) was not associated 25(OH)D concentrations (*P* = 0.57), neither were the extended HLA-groups (nonincreased risk, slightly increased risk, moderately increased risk, and highly increased risk in HLA-DR/DQ) (*P* = 0.46).

The age of the mother at the time of birth was positively associated with 25(OH)D concentration (*P* = 0.002) cord serum with higher concentration in older mothers. The median age of the mother was 29.8 (IQR 26.6 to 33.6) years, and it was highest in Turku [30.2 (IQR 26.6 to 33.7) years] and lowest in Oulu [29.4 (26.3 to 33.0) years]. The ponderal index of the child, calculated as weight in kilogram per height cubed, was inversely associated with 25(OH)D concentrations (*P* = 0.031), but there was no statistically significant association with either weight (*P* = 0.29) or length (*P* = 0.75) alone to 25(OH)D. The ponderal index did not correlate with the age of the mother (*P* = 0.16).

Almost 10% of the mothers had smoked during pregnancy (70 smoking, 655 nonsmoking, 39 not known). There were mothers who had smoked in all groups: 20 were mothers to T1D+ children, 26 to T1D− children, 9 to IA+ children, and 15 to IA−children. Maternal smoking (yes/no) during pregnancy was not associated with 25(OH)D concentration in cord serum (*P* = 0.40) and neither was the mode of delivery when all modes (vaginal delivery, emergency cesarean section, planned cesarean section, or assisted delivery) were taken into account (*P* = 0.17).

Because some have found cord serum 25(OH)D concentration quartiles to be an important factor in relation to T1D odds (14), we decided to analyze those as well. However, when we divided the samples into quartiles based on the 25(OH)D concentration (Table 2), the overall difference between the groups (T1D+, T1D−, IA+, and IA−) remained statistically nonsignificant (*P* = 0.29), whereas study center and season remained statistically significant (*P* = 0.003 and *P* < 0.001, respectively).

**Table 2.** Subjects Divided Into Quarters Based on 25(OH)D Concentration

	Q1 (n = 191)	Q2 (n = 191)	Q3 (n = 191)	Q4 (n = 191)	*P*
	20.14 (9.40–24.00)^*a*^	27.75 (24.00–31.06)^*a*^	34.95 (31.06–41.72)^*a*^	49.27 (41.76–135.1)^*a*^	
T1D+ / T1D− / IA+ / IA-	74 / 55 / 35 / 27	52 / 69 / 34 / 36	55 / 70 / 30 / 36	69 / 56 / 33 / 33	0.29
Turku / Oulu / Tampere	51 / 91 / 49	64 / 69 /58	74 / 62 / 55	77 / 52 / 62	**0.003**
Season (January-March)/ (April-June) / (July-September) / (October-December)	74 / 53 / 10 / 54	65 / 54 / 22 / 50	54 / 46 / 43 / 48	17 / 29 / 112 / 33	**<0.001**
Girls / boys	82 / 109	70 / 121	81 / 110	77 / 114	0.58
Old HLA high/moderate	50 / 141	68 / 123	64 / 127	64 / 127	0.20
New HLA (NA/nonincreased slight/moderate/high)	34 / 9 / 40 / 70 / 37	27 / 8 / 33 / 80 / 43	51 / 11 / 29 / 52 / 48	26 / 7 / 33 / 77 / 48	**0.027**
Median ponderal index, kg/m^3^ (IQR)	27.84 (26.31–29.60)	27.71 (26.37–29.44)	27.59 (25.88–29.11)	27.55 (25.71–29.10)	0.12
Median weight, kg (IQR)	3.61 (3.28–3.95)	3.68 (3.36–3.97)	3.63 (3.28–3.93)	3.53 (3.27–3.90)	0.28
Median height, cm (IQR)	51 (49–52)	51 (49–52)	51 (49–52)	50 (49–52)	0.77
Mothers nonsmoking/smoking/NA	160 / 21 / 10	163 / 15 / 10	170 / 17 / 4	162 / 14 / 15	0.70
Cases with IAA/GAD appearing first	31 / 26	28 / 31	34 / 19	30 / 33	0.25
Children with 0/1/2/3/4 autoantibodies	81 / 2 / 20 / 28 / 55	105 / 1 / 23 / 22 / 38	106 / 2 / 16 / 26 / 40	88 / 1 / 19 / 33 / 44	0.37

The value of *P* < 0.05 (two-tailed) was taken to indicate statistical significance and is marked in bold.

^*a*^Median 25(OH)D (range), nmol/L.

### Nutrition during pregnancy

We were able to obtain nutritional information from the eighth month of pregnancy in almost half (48%) of the study population, as 61% of the Oulu and 87% of the Tampere cohorts participated in the DIPP Nutrition Study. The general characteristics of the subpopulation for which nutritional information was available are presented in Table 3.

**Table 3.** General Characteristics of the Subpopulation Participating in Nutritional Study During Pregnancy

Group	T1D+ n = 121	T1D− n = 119	IA+ n = 63	IA− n = 60	*P*
Oulu/Tampere	51 / 70	49 / 70	35 / 28	33 / 27	0.11
Mother of a boy/girl	54 / 67	52 / 67	26 / 37	24 / 36	0.94
Age of mother, y (median [IQR])	29.6 (26.5–34.2)	29.3 (26.7–33.2)	28.4 (25.2–32.7)	29.1 (26.8–33.0)	0.76
Birth weight, kg (median [IQR])	3.5 (3.2–3.9)	3.7 (3.3–4.0)	3.6 (3.2–3.9)	3.6 (3.3–4.0)	0.56
Birth height, m (median [IQR])	0.50 (0.49–0.52)	0.51 (0.49–0.52)	0.51 (0.49–0.52)	0.50 (0.49–0.52)	0.81
Ponderal index, kg/m^3^ (median [IQR])	27.9 (26.4–29.5)	28.3 (26.3–29.5)	27.7 (26.1–28.7)	27.7 (26.4–29.6)	0.34
Energy intake per day, kJ (median [IQR])	10,876 (8842–13,062)	10,876 (9808–13,094)	11,364 (9666–12,976)	11,354 (9364–12,656)	0.38
Vitamin D intake per day from food, µg (median [IQR])	4.92 (3.47–6.69)	5.24 (3.75–7.74)	5.22 (3.45–7.01)	4.71 (2.94–6.51)	0.21
Vitamin D intake per day from supplements, µg (median [IQR])	0 (0–2.11)	0 (0–1.5)	0 (0–2.39)	0 (0–0.76)	0.85
Total vitamin D intake per day, µg (median [IQR])	5.84 (3.91–8.96)	6.53 (4.06–9.84)	5.96 (4.09–9.29)	6.53 (4.06–9.84)	0.38

The value of *P* < 0.05 (two-tailed) was taken to indicate statistical significance.

Maternal intake of vitamin D during pregnancy showed a statistically significant association with umbilical cord serum 25(OH)D concentrations in all forms, from food (*P* < 0.0001), from supplements (*P* < 0.0001), and in total (*P* < 0.0001). The energy intake was not associated with 25(OH)D concentration (*P* = 0.27).

The age of the mother had a statistically significant association with 25(OH)D concentration also in this subpopulation (*P* = 0.025), but age did not correlate with energy intake (*P* = 0.64), vitamin D intake from food (*P* = 0.22), intake from supplements (*P* = 0.51), nor with total vitamin D intake (*P* = 0.22). The median age of mothers was in the same range [29.3 years (IQR 26.4 to 33.4)] in this subpopulation as it was in the entire study cohort [29.8 years (IQR 26.6 to 33.6)].

Ponderal index of the infant was not associated with the 25(OH) concentration in this subpopulation (*P* = 0.097) but its correlation with the age of the mother was close to significant (*P* = 0.059). Ponderal index did not correlate with energy intake (*P* = 0.34), vitamin D intake from food (*P* = 0.30), from supplements (*P* = 0.52), nor with total vitamin D intake (*P* = 0.93) of the mother during pregnancy.

When divided into the same quartiles based on 25(OH)D quarters as the entire data set (Table 4), there were no statistically significant differences in the proportion of children participating, or in energy intake in each quartile, but there were differences in vitamin D intake from food (*P* < 0.001), from supplements (*P* < 0.001), and in total (*P* < 0.001).

**Table 4.** Nutritional Data During the Eighth Month of Pregnancy When Divided Into the Same 25(OH)D Concentration Quarters as the Entire Data Set in Table 2

	Q1	Q2	Q3	Q4
	20.14 (9.40–24.00)^*a*^	27.75 (24.00–31.06)^*a*^	34.95 (31.06–41.72)^*a*^	49.27 (41.76–135.1)^*a*^	*P*
Median 25(OH)D (range) of subcohort, nmol/L	20.53 (9.40–24.00)	27.76 (24.00–31.06)	35.12 (31.11–41.72)	50.06 (41.76-105.1)	
N of children with nutritional data during pregnancy	103	87	88	85	0.23
Vitamin D intake from food per day, µg (median [IQR])	4.25 (2.95–5.86)	4.83 (3.54–6.84)	4.94 (3.58–6.95)	6.43 (4.29–9.25)	**0.007**
Vitamin D intake from supplements per day, µg (median [IQR])	0 (0–0)	0 (0–1.50)	0 (0–3.99)	0 (0–2.97)	**<0.001**
Total vitamin D intake per day, µg (median [IQR])	4.76 (3.15–6.10)	5.91 (3.72–8.62)	7.26 (4.16–9.17)	8.43 (5.09–12.00)	**<0.001**
Energy intake per day, kJ (median [IQR])	11,107 (9424–12,976)	11,051 (8922–13,789)	11,220 (8905–13,298)	11,617 ( 10,000–13,490)	0.71

The value of *P* < 0.05 (two-tailed) was taken to indicate statistical significance and is marked in bold.

^*a*^Original median 25(OH)D (range), nmol/L.

The total vitamin D intake during pregnancy was quite low, as there were only 72 children (20%) in this cohort whose mothers reported a total vitamin D intake during pregnancy ≥10 µg per day; 22 T1D+, 29 T1D−, 14 IA+, and 7 IA−. The median 25(OH)D concentration in these 72 serum samples was 39.1 (IQR 30.1 to 46.6) nmol/L.

The use of vitamin D supplementation during pregnancy was uncommon, with only 33 mothers (9%) getting ≥5 µg vitamin D from supplements.; 10 T1D+, 13 T1D−, 6 IA+, and 4 IA−. The supplement use was equally distributed between seasons; nine in winter, seven in spring, nine in summer, and eight in fall. Because the nutritional information was based on the eighth month of pregnancy, the season was mostly the same as the season of birth.

## Discussion

Based on this study, fetal vitamin D status, reflected by umbilical cord serum 25(OH)D (15–17), does not appear to have an association with IA or with progression to T1D. The 25(OH)D concentrations of the pregnant mothers are lower than in the children in the upcoming months and years (2). The levels in cord serum or plasma seemed to be low worldwide (18, 19) until recently (17).

Vitamin D supplementation is recommended for the whole growth period for all children in Finland and the parents comply well with this recommendation, at least for the first few years of life (20). The conspicuous monthly variation and the overall low 25(OH)D concentrations alone suggest that the pregnant women have not been taking commonly vitamin D supplementation, and this was confirmed by the nutritional data available. Similarly, in an earlier study of 1669 mothers from the same population cohort in Tampere and Oulu, the mean maternal intake of vitamin D was 5.1 (SD 2.6) µg from food and 1.4 (SD 2.6) µg from supplements, with only 32% of women taking vitamin D supplements during approximately the same study period, *i.e.,* years 1997 to 2001 (21). It is noteworthy that we found no seasonal variation in vitamin D supplement usage, even though the recommendations advised pregnant women to use supplements only during the winter months (22–24). Thus, it seems that most Finnish mothers did not take care of their own vitamin D supplementation during pregnancy at the time but were careful in supplementation of their children.

The major strength of the current study is that it includes a large number of children in matched case control pairs and there is abundant follow-up data on these children. Collecting samples for more than a decade is not an easy task to accomplish but it helps minimize annual fluctuations.

Some study limitations should also be considered. One is that the children were originally selected for the DIPP study based on their HLA-DQB1 genotype and we did not have any genetic information on the mothers. No association of HLA-DRB1 or HLA-DQB1 with 25(OH)D concentrations has been reported to the best of our knowledge, but an association of cord serum 25(OH)D concentrations and maternal HLA-B44 (25) and two single nucleotide polymorphisms, one for the vitamin D receptor gene and one for the group-specific component gene (26), have been observed in Finnish studies. Furthermore, we did not have single nucleotide polymorphisms of the vitamin D pathway in the children, which might have been interesting to have, as some of us, participating in a large international collaboration study, found that 25(OH)D levels and vitamin D receptor polymorphism may have a combined role in the development of islet autoimmunity in children at increased genetic risk for T1D (27).

It is noteworthy that the case children in this study were born evenly around the year in both case groups, those who developed T1D and those remaining autoantibody positive. This alone should indicate that the 25(OH)D concentration at birth may not be as important in the development of T1D as previously suggested (14). In an updated analysis of the same individuals of that previous Norwegian study, no association between first and second trimester 25(OH)D concentrations and childhood risk of T1D was observed (28). By analyzing neonatal blood spots, a small Italian study found no association between 25(OH)D levels and risk of T1D, except in a subgroup of migrant infants (29). A Danish research group showed with a larger cohort that that neonatal 25(OH)D status was not associated with a later risk of T1D (30). We were able to verify those results and went further, as we showed for the first time that 25(OH)D levels at birth were not associated with the progression to clinical T1D, the number of islet autoantibodies, nor with which autoantibodies appear first.

This and the other studies in which no differences regarding later risk of T1D was observed (10, 11), had very low median 25(OH)D levels. It might be possible that a difference could be detected in a more vitamin D sufficient population. However, in another autoimmune disease, multiple sclerosis, it was the lower spectrum of levels where the risk of the disease was most prominent (12). Accordingly it is unlikely that there is any detectable difference in relation to T1D, which was further supported by our finding that no difference were seen even in the highest 25(OH)D concentration quartile where the median levels were very close to normal.

Cord serum 25(OH)D concentrations may have a long-term influence on later health and associations have been reported with childhood metabolic profiles (31) and early childhood growth together with neural development (32). Maternal vitamin D levels have been shown to have more influence on cord serum 25(OH)D than the genetic factors of the offspring (16). As we showed here, maternal intake of vitamin D during pregnancy has a clear impact on cord serum 25(OH)D levels and quite surprisingly, vitamin D intake was not associated with energy intake or maternal age. The factors behind the higher 25(OH)D concentrations in the cord serum samples of older mothers, which has been reported also in other studies (33, 34), would require further research.

## Abbreviations:

25(OH)D: 25-hydroxyvitamin D
DIPP: Type 1 Diabetes Prediction and Prevention study
GADA: glutamic acid decarboxylase autoantibody
HLA: human leukocyte antigen
IA: islet autoimmunity
IAA: insulin autoantibody
IA-2A: autoantibody against the tyrosine phosphatase-related IA-2 protein
ICA: islet cell antibody
IQR: interquartile range
T1D: type 1 diabetes
